# Evaluation of Lightning Strike Fatalities: A Retrospective Autopsy Study from 2 Centers in Eastern Türkiye

**DOI:** 10.5152/eurasianjmed.2026.261359

**Published:** 2026-06-11

**Authors:** Abdulkadir Sancı, Eniscan Karaalp, Ayşe Seydaoğulları Baltacı, Talip Vural

**Affiliations:** 1 Department of Forensic Medicine, Kars Forensic Medicine Branch Directorate, Kars, Türkiye; 2 Department of Forensic Medicine, İzmir Forensic Medicine Group Presidency, İzmir, Türkiye; 3 Department of Forensic Medicine, Ardahan Forensic Medicine Branch Directorate, Ardahan, Türkiye; 4 Department of Forensic Medicine, Atatürk University Faculty of Medicine, Erzurum, Türkiye

**Keywords:** Forensic autopsy, Lichtenberg figures, lightning strike, scene investigation

## Abstract

**Background:**

Deaths due to lightning strikes are rare but of great forensic importance because of their sudden and fatal nature. This study aimed to evaluate the demographic characteristics, scene findings, and autopsy results of fatal lightning strike cases examined in Kars and Ardahan provinces.

**Methods:**

This retrospective study included 13 cases of death by lightning strike, identified from 847 autopsies performed in 2 climatically similar provinces between 2019 and 2025 via the National Judicial Network Project (UYAP) database. The research evaluates the victims’ demographic data, incident location characteristics, and macroscopic autopsy findings, alongside negative toxicological and histopathological examination results. The data were analyzed using the SPSS software, utilizing descriptive statistical methods such as frequency, percentage distribution, and mean values.

**Results:**

Between 2019 and 2025, 1.53% (n = 13) of forensic autopsies in Kars and Ardahan were attributed to lightning strikes, with all cases involving male victims and a mean age of 31.3 years. The majority of cases were shepherds (69.2%), and deaths occurred most frequently in rural pastures during the spring and summer months, particularly in May and June. While all cases exhibited first- and second-degree burns and singed body hair, characteristic Lichtenberg figures were detected in 46.1% of the victims alongside various internal hemorrhages in some instances. Crime scene investigations provided critical diagnostic evidence, including the presence of deceased livestock near the victims and partially burned or torn personal belongings.

**Conclusion:**

Lightning-related deaths show a strong association with seasonal, occupational, and environmental factors. Scene investigation and the recognition of Lichtenberg figures play a crucial role in the forensic diagnosis of lightning strike fatalities.

**Main Points**

- All victims were male, and most deaths occurred during spring and summer seasons, predominantly in open rural areas among shepherds.
- The most common autopsy findings were singeing of body hair and Lichtenberg figures.
- Scene investigation and the recognition of Lichtenberg figures play a crucial role in the forensic diagnosis of lightning strike fatalities.

## Introduction

Lightning occurs when a circuit is completed between the base of a storm cloud and the earth’s surface.^1^ It is the second most common weather-related natural event causing fatalities, following floods.^2^ The energy released by lightning is approximately 30 million volts, with an intensity of 30 000 to 50 000 amperes. During a lightning strike, temperatures can reach around 30 000 degrees Celsius, and the pressure can be as high as 100 atmospheres.^3^ Between 10% and 30% of injuries related to lightning strikes result in death.^4^ A study conducted in Türkiye found that 0.7% of forensic autopsies were attributed to lightning strike fatalities.^5^

Death from lightning strikes is typically caused by cardiac arrhythmias and asystole.^6^ While there may be no visible external signs on the body, burns, electrical entry and exit wounds, or torn clothing may be present.^7^ To determine whether a person found dead in an open area succumbed to a lightning strike, a comprehensive internal and external examination of the deceased is essential, along with a thorough investigation of the scene. Factors to consider include whether the weather was rainy and stormy at the time of the incident, the presence of scattered pieces of clothing in the vicinity, damage to nearby houses and trees, and any mass animal deaths.^8^ Shredded or burned clothing, as well as magnetized or melted metal objects found on the person, can provide crucial insights, especially since there may be no specific signs in the internal organs to assist in diagnosis during the autopsy, aside from Lichtenberg figures and burn lesions on the skin.^9^

This study aimed to evaluate the autopsy findings of cases determined to have died from lightning strikes. The concentration of storms in high-altitude, wide-open areas often leaves those engaged in agriculture and livestock farming exposed for long periods, with limited access to safe shelter. The autopsies were performed at the Kars and Ardahan Forensic Medicine Branch Directorates of the Forensic Medicine Institute. This is one of the few forensic studies in Türkiye evaluating lightning-related deaths with a combined assessment of autopsy and crime scene findings in 2 neighboring high-altitude provinces.

## Material and Methods

This study was conducted through a retrospective analysis of 847 autopsy reports performed between January 1, 2019, and September 1, 2025, at the Ministry of Justice Kars and Ardahan Forensic Medicine Branch Directorates, located in 2 neighboring provinces with similar climatic and geographical characteristics. The research data were filtered through the National Judicial Network Project (UYAP) database using the keyword “lightning” (yıldırım), identifying 13 cases where the cause of death was recorded as a lightning strike. All causes of death other than lightning strikes were excluded from the study as part of the exclusion criteria.

For the 13 cases included in the study, demographic data (age, gender, and occupational groups), locality data (geographical region and seasonal characteristics), and postmortem findings, including macroscopic autopsy findings and injury patterns, were analyzed. Furthermore, toxicological and histopathological examination results were evaluated. It was confirmed that histopathological sampling was performed in all cases, and no toxic substances (negative toxicological findings) capable of explaining the death were detected. Statistical analysis was performed using the IBM SPSS Statistics for Windows, Version 22.0 (IBM SPSS Corp.; Armonk, NY, USA). The study is descriptive in nature, employing statistical methods such as frequency, percentage distribution, and mean values for data presentation. Because of the limited number of cases, only descriptive statistical methods were applied.

Ethics committee approval for the study was obtained from the Education and Scientific Research Commission of Ministry of Justice Forensic Medicine (date: 11.11.2025, decision no: 2025/1441). All methods were performed in accordance with the relevant guidelines and regulations outlined in the Declaration of Helsinki. Informed consent was waived by the Ethics committee due to the retrospective nature of the study.

## Results

Out of 847 forensic autopsies conducted at the Kars and Ardahan Branch Directorates of the Ministry of Justice Forensic Medicine Institute between January 1, 2019, and September 1, 2025, it was determined that 1.53% (n = 13) of the deaths were due to lightning strikes. All 13 cases included in the study (100%) were male (Table 1). The ages of the cases ranged from 7 to 69 years, with a calculated mean age of 31.3. Regarding occupational distribution, the vast majority of the cases (69.2%, n = 9) were shepherds, while the remaining 4 cases (30.8%) were unemployed (pediatric/adolescent age group).

When the seasonal distribution of the deaths was examined, it was found that the incidents occurred most frequently in the spring (38.4%, n = 5) and summer (46.1%, n = 6), with 2 cases (15.3%) recorded in the autumn. On a monthly basis, the highest density was observed in May (n = 4) and June (n = 2). In terms of the location of death, 10 cases (76.9%) died at the scene (rural area/pasture), while 3 cases (23.1%) died after being transported to the hospital.

**Figure 1.**
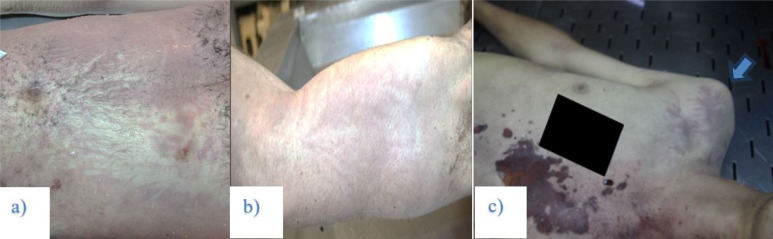
External examination findings from different cases. (a) Lichtenberg figure in the abdominal region. (b) Lichtenberg figure on the right arm. (c) Burn lesions in the chest region and Lichtenberg figure on the right shoulder (blue arrow).

First- and second-degree burns were detected in all cases (100%) (Table 2). The most common finding in the external examination was the singeing of body hair, observed in almost all cases. Lichtenberg figures, a characteristic finding of lightning strikes, were detected in 6 of the 13 cases (46.1%) (Figure 1). These figures were localized on the abdomen, chest, right arm, and left thigh. Internal examination revealed subarachnoid hemorrhage in 3 cases (23%), pulmonary hemorrhage in 2 cases, liver laceration in 1 case, and liver hemorrhage in 1 case. In the majority of the cases (53.8%, n = 7), no specific macroscopic pathological findings were found in the internal organs.

**Figure 2.**
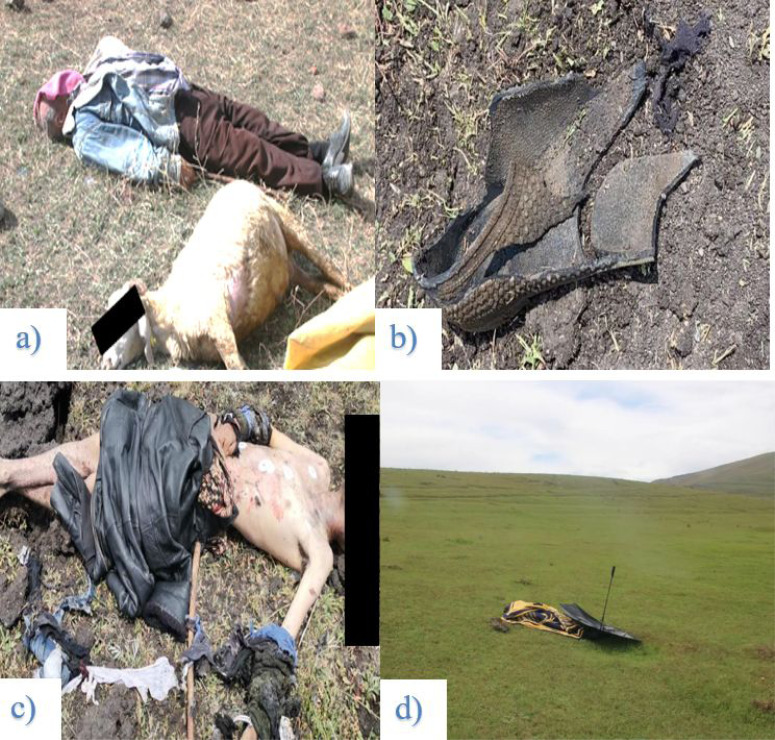
Crime scene findings from different cases. (a) Dead sheep next to the body at the crime scene. (b) The body’s torn-up shoes at the crime scene. (c) Partially burnt and torn-up clothes on the body at the crime scene. (d) Umbrella found next to the body at the scene of death.

Upon examining the crime scene investigation findings of different cases, a dead sheep was found next to the body of one victim, and partially burned and torn personal belongings were found in 3 cases (Figure 2).

## Discussion

Approximately 100 lightning strikes occur every second worldwide.^10^ According to the National Oceanic and Atmospheric Administration, a total of 220 deaths due to lightning strikes were reported in the United States between 2015 and 2025. These data position lightning as the second most common weather-related cause of death after floods.^11^^-^^14^ In national studies, lightning-related deaths have been reported to account for 0.7% of autopsy cases in Diyarbakır, 0.27% in Van, and 0.4% across the Eastern Anatolia region. Globally, the mortality rate associated with lightning strikes is estimated to range between 0.2 and 1.7 per million people.^15^^,^^16^ In this study, however, 1.53% of the autopsied cases were determined to have died as a result of lightning strikes. These findings suggest that the mortality rate identified in the study is relatively higher when compared with both national and global data. This difference may be attributed to the geographical and climatic characteristics of the provinces of Kars and Ardahan, including their extensive high-altitude plains and the substantial precipitation that begins in April and continues throughout the summer.^17^^,^^18^

Individuals most frequently exposed to lightning strikes are those who work outdoors or participate in outdoor activities, such as campers, hikers, farmers, construction workers, golfers, and hunters.^6^ In eastern Türkiye, lightning-related deaths are observed more frequently than in other regions, as agriculture and livestock are primary sources of income.^16^^,^^19^ Since outdoor activities are more commonly performed during the spring and summer months, and precipitation is more frequent during these periods, the incidence of lightning strikes tends to parallel the frequency of lightning activity. In this context, lightning-related injuries are expected to occur most frequently in June, July, August, and September.^20^^,^^21^

Research conducted across different regions of Türkiye demonstrates a clear correlation between lightning-related fatalities and outdoor agricultural activities, albeit with varying intensities. A study in Eskişehir (1997-2011) revealed that 75% of cases involved farmers or shepherds. This occupational concentration remains high in the Black Sea region; a 10-year retrospective study in Trabzon reported that 79% of victims belonged to these groups. In the rural and high-altitude landscape of Van, this figure reached 100%. Conversely, in highly urbanized Istanbul, the proportion of farmers and shepherds among the deceased was significantly lower, accounting for 46.15% of total cases.^21^^,^^22^ In this study, the findings that most cases consisted of adult male shepherds, incidents occurred predominantly during the spring and summer, and deaths occurred without medical intervention are consistent with this established pattern. This observation suggests that lightning strikes represent both an occupational and seasonal risk factor, particularly for adult males engaged in livestock farming in open areas.

The literature indicates that a person struck by lightning is exposed to light, heat, electrical, and pressure forces, and injuries are reported to develop under the influence of these forces. It has been reported that victims may experience multisystem disorders, with prominent effects observed in the cardiovascular and central nervous systems.^23^ Consistent with literature data, the fact that death occurred at the scene rather than in a hospital in the majority of the cases may be associated with the rapid progression of conditions such as cardiopulmonary arrest. Furthermore, literature suggests that the pressure effect may be associated with pulmonary findings such as lung contusions, pneumothorax, and acute respiratory distress syndrome, as well as neurological damage and injuries to the spleen, liver, and bowel.^24^^,^^25^

Such internal organ findings may be revealed during internal examination at autopsy, while external examination may show no visible signs of these injuries. On external examination, extensive burns, charring of hair, and lesions known as “Lichtenberg figures” may be observed.^6^ A study in Trabzon reported macroscopic findings indicative of electrical entry in all cases and Lichtenberg figures in approximately half of the cases.^22^ In the study, singed body hair was observed during external examination in all cases, whereas Lichtenberg figures and internal organ pathology were present in more than half of the cases. The consistent observation of singed body hair across all cases suggests it may be one of the reliable findings during the external examination of lightning strikes. Lichtenberg figures were observed in a subset of cases and represent characteristic external findings in lightning-related deaths.

In the study, lacerations and hematomas were detected in the heart, brain, lungs, and esophagus, whereas traumatic bone injuries were observed less frequently. Internal findings such as hemorrhage and laceration were observed in some cases; although non-specific, these findings may be associated with lightning exposure when interpreted together with scene and external examination findings.^26^ Forensic literature on deaths related to electrical injuries emphasizes that internal organ findings are generally non-specific and that establishing a definitive mechanism based solely on internal pathology—without external markers or scene data—is challenging. The high-voltage but brief nature of lightning can result in death without leaving significant thermal damage; this clearly highlights the limitations of external findings and the necessity of a holistic, multidisciplinary evaluation.

A thorough scene investigation is of critical importance in the investigation of lightning strikes. Scattered clothing, damage to buildings and trees, or mass animal deaths are significant pieces of evidence.^27^^,^^28^ In the study, burning and tearing of clothing were observed in a subset of cases, and in 1 instance, a dead sheep was found next to the body. The absence of specific environmental findings or clothing damage in some cases suggests that relying on a single parameter for diagnosis may be insufficient. The low incidence of clothing damage could be attributed to the brief passage of current over the body surface (flashover) or to environmental factors obscuring evidence. Therefore, lightning strike incidents should be approached as a multidisciplinary effort, where external and internal examination findings are interpreted holistically along with forensic data.

The limitations of this study include the small sample size and the retrospective design, which prevented access to detailed meteorological data at the time of the incidents.

In conclusion, the study demonstrates that deaths resulting from lightning strikes exhibit a distinct seasonal and occupational distribution. The majority of cases occur during the spring and summer months, predominantly affecting males working in open areas (shepherds). These findings may be interpreted in the context of lightning-related injury. Furthermore, the high proportion of incidents occurring in open terrain without witnesses underscores the critical importance of examining autopsy and scene findings together in forensic evaluations. Based on the data obtained, essential strategies to reduce preventable deaths include the effective use of meteorological warning systems, the construction of safe shelters, and the enhancement of regional disaster awareness.

## Figures and Tables

**Table 1.** Demographic Characteristics of the Cases

**Case**	**Gender**	**Age**	**Year**	**Month**	**Season**	**Profession**	**Where the Death Occurred**
1	Male	16	2023	6	Summer	Not working	Rural
2	Male	66	2019	11	Autumn	Shepherd	Rural
3	Male	46	2020	7	Summer	Shepherd	Rural
4	Male	27	2021	4	Spring	Shepherd	Rural
5	Male	14	2024	5	Spring	Not working	Rural
6	Male	20	2025	5	Spring	Shepherd	Rural
7	Male	69	2020	9	Autumn	Shepherd	Hospital
8	Male	7	2021	6	Summer	Not working	Rural
9	Male	57	2021	8	Summer	Shepherd	Rural
10	Male	23	2023	5	Spring	Shepherd	Hospital
11	Male	19	2023	4	Spring	Shepherd	Hospital
12	Male	15	2024	8	Summer	Not working	Rural
13	Male	69	2021	9	Autumn	Shepherd	Rural

**Table 2.** Examination Findings and Internal Organ Injuries of the Cases

**Case**	**Burn Degree**	**External Examination Findings**	**Internal Organ Findings**
1	1 and 2	Singeing of body hair	Subarachnoid hemorrhage
		Lichtenberg figure on the right arm	Pulmonary hemorrhage
		Skin burn on the right upper extremity	
2	1 and 2	Singeing of body hair	None
3	1 and 2	Singeing of body hair	Subarachnoid hemorrhage
4	1 and 2	Singeing of body hair	None
		Lichtenberg figure on the abdomen	
5	1 and 2	Singeing of body hair	Subarachnoid hemorrhage
6	1 and 2	Singeing of body hair	None
7	1 and 2	Singeing of body hair	Liver laceration
		Lichtenberg figure on the chest	
8	1 and 2	Lichtenberg figure on the left thigh	None
		Avulsion of the left auricle (earlobe)	
9	1 and 2	Singeing of body hair	Liver hemorrhage
		Lichtenberg figure on the abdomen	
10	1 and 2	Singeing of body hair	None
11	1 and 2	Singeing of body hair	None
12	1 and 2	Singeing of body hair	None
13	1 and 2	Singeing of body hair	Pulmonary hemorrhage
